# Enhanced syphilis screening among HIV-positive men (ESSAHM): a study protocol for a clinic-randomized trial with stepped wedge design

**DOI:** 10.1186/s13012-016-0371-0

**Published:** 2016-01-16

**Authors:** Ann N. Burchell, Vanessa G. Allen, Ramandip Grewal, Paul A. MacPherson, Anita Rachlis, Sharon Walmsley, Sharmistha Mishra, Sandra L. Gardner, Janet Raboud, Curtis Cooper, Kevin Gough, Sean B. Rourke, Rodney Rousseau, Irving Salit, Darrell H. S. Tan

**Affiliations:** 1 Department of Family and Community Medicine, St. Michael’s Hospital, 30 Bond Street, Toronto, Ontario M5B 1W8 Canada; 2 Centre for Research on Inner City Health, Li Ka Shing Knowledge Institute, St. Michael’s Hospital, Toronto, Canada; 3 Dalla Lana School of Public Health, University of Toronto, Toronto, Canada; 4 Public Health Ontario Laboratories, Public Health Ontario, Toronto, Canada; 5 Division of Infectious Diseases, The Ottawa Hospital, Ottawa, Canada; 6 Ottawa Hospital Research Institute, Ottawa, Canada; 7 Department of Medicine, University of Ottawa, Ottawa, Canada; 8 Sunnybrook Health Sciences Centre, Toronto, Canada; 9 Department of Medicine, University of Toronto, Toronto, Canada; 10 Toronto General Hospital, University Health Network, Toronto, Canada; 11 Li Ka Shing Knowledge Institute, St. Michael’s Hospital, 30 Bond Street, Toronto, Ontario M5B 1W8 Canada; 12 Division of Infectious Diseases, Department of Medicine, University of Toronto, Toronto, Canada; 13 Ontario HIV Treatment Network, Toronto, Canada; 14 Toronto General Research Institute, University Health Network, Toronto, Canada; 15 Division of Infectious Diseases, St. Michael’s Hospital, Toronto, Canada; 16 Department of Psychiatry, University of Toronto, Toronto, Canada; 17 Department of Immunology, University of Toronto, Toronto, Canada; 18 Poz Prevention Working Group, Gay Men’s Sexual Health Alliance, Toronto, Canada

**Keywords:** HIV, Syphilis, Men, Screening, Outpatient clinics, Intervention, Cluster-randomized controlled trial

## Abstract

**Background:**

The current syphilis epidemic among urban men who have sex with men (MSM) has serious implications for those co-infected with human immunodeficiency virus (HIV). Routine and frequent syphilis screening has the potential to ensure early detection and treatment, minimize disease burden, and help control the ongoing spread of syphilis and HIV. We aim to enhance syphilis screening among HIV-positive men by conducting a clinic-based intervention that incorporates opt-out syphilis testing into routine HIV laboratory evaluation for this population. Trial objectives are to determine the degree to which the intervention (1) increases the detection rate of untreated syphilis, (2) increases screening coverage, (3) increases screening frequency, and (4) reaches men at highest risk according to sexual behaviors.

**Methods/design:**

The trial is a pragmatic, stepped wedge cluster-randomized controlled trial that introduces the intervention stepwise across four urban HIV clinics in Ontario, Canada. The intervention includes standing orders for syphilis serological testing whenever a male in HIV care undergoes HIV viral load testing, which typically occurs every 3–6 months. The control condition is the maintenance of current, provider-initiated syphilis testing practice. Approximately 3100 HIV-positive men will be followed over 30 months. Test results will be obtained from the centralized provincial laboratory in Ontario and will be supplemented by a standardized clinical worksheet and medical chart review at the clinics. Detailed clinical, psychosocial, and behavioral data is available for a subset of men receiving HIV care who are also participants of the province-wide Ontario HIV Treatment Network Cohort Study. Process evaluation plans include audit and feedback of compliance of the participating centers to identify potential barriers to the introduction of this type of practice into routine care. Health economic components include evaluation of the impact and cost-effectiveness of the intervention.

**Discussion:**

This trial will be the first of its kind in Canada and will provide evidence regarding the feasibility, clinical effectiveness, and cost-effectiveness of a clinic-based intervention to improve syphilis screening among HIV-positive men. Involvement of knowledge users in all stages of trial design, conduct, and analysis will facilitate scale-up should the intervention be effective.

**Trial Registration:**

ClinicalTrials.gov NCT02019043

## Background

After years of decline, in many urban settings internationally, including Canada, syphilis has re-emerged as a serious health burden among men who have sex with men (MSM) [1–5]. Syphilis is caused by the bacterium *Treponema pallidum*. Transmission occurs from an active case via sexual activity involving oral, vaginal, or anal mucosae [6]. For men who are co-infected with human immunodeficiency virus (HIV), transmission rates may be increased, the response to treatment can be suboptimal [7, 8], the development of neurosyphilis may be accelerated [9, 10], treatment decisions are more complex, and HIV infectiousness may be enhanced [6, 11–13]. In Ontario, lifetime syphilis prevalence among MSM in HIV care was estimated to be 23.4 % as of 2009, and in 2010, incidence of a new syphilis diagnosis was 4.3 per 100 person-years (PY), over 300 times greater than the rate of 0.01 per 100 PY reported for the general male population [14]. Repeat diagnoses (reflecting repeat infections) of syphilis were higher at 4.8 per 100 PY (95 % 3.7, 5.5). These rates are under-estimates of true prevalence and incidence because they reflect cases detected by routine practice rather than by active screening programs.

Frequent syphilis screening has the potential to ensure early detection and treatment of cases, minimize disease burden for patients, and help control the ongoing spread of syphilis and HIV [15, 16]. International guidelines recommend syphilis screening among people with HIV at least once per year, with some advising more frequent testing at 3- to 6-month intervals [6, 17, 18]. Canadian Sexually Transmitted Infection (STI) Guidelines recommend screening for syphilis, gonorrhea, chlamydia, and HIV at 3-month intervals even in the absence of symptoms—for individuals identified at “ongoing risk for STIs” [6]. Preliminary mathematical models suggest that frequent syphilis screening, at minimum twice per year, could have substantial impacts on reducing syphilis transmission among the most high-risk MSM [16, 19, 20].

Despite the guidelines, current levels of screening and testing remain infrequent in many settings [21–23]. Screening is often limited to opportunistic screening when men self-disclose sexual risk behavior. Diagnostic tests are restricted to those presenting with signs or symptoms and miss asymptomatic or mild cases in persons who may not seek medical attention. For example, in a nationally representative sample of persons in HIV care in the USA, only 55 % of sexually active patients were tested for syphilis at least once per year [21]. In Ontario in 2009, 53 % of HIV-positive MSM received syphilis screening or testing on average once per year [22]. As of 2013, we observed only a modest increase in this proportion to 64 % screened annually (unpublished data). Although in Ontario we have observed that HIV-positive men reporting risk behavior are more likely to be tested, there is room for improvement; for example, among men who reported five or more sexual partners in the preceding 3 months, 22 % were not tested for syphilis over the subsequent 2-year period in 2010–2013 [23].

Evidence from uncontrolled trials and observational studies suggests that clinic-based, opt-out interventions may improve syphilis screening coverage and case detection [24–27]. With men in HIV care, there is a practical and relatively inexpensive opportunity to intervene because patients undergo routine blood tests for HIV viral load every 3–6 months [28, 29]. An outpatient HIV clinic in London, UK, added syphilis serology to computerized routine blood order sets. Compared to a 12-month pre-intervention period, this intervention led to an increase from 3 to 85 % of patients receiving at least one syphilis test, a reduction in the median time between syphilis tests from 6 to 4 months, and a 27 % increase in the number of early syphilis cases detected during the 12-month intervention period [24]. An anal cytology screening program in an urban Australian HIV clinic for HIV-positive MSM offered joint STI testing, which increased bacterial STI testing (including syphilis) from 20 to 35 % over 3 months, and they diagnosed four new cases compared to none previously [25]. In 2007, the Melbourne Sexual Health Centre in Australia implemented automatic stamping of syphilis serology orders on all HIV laboratory request forms [26]. Post-intervention, the median number of tests per patient per year rose from 1 to 2. There were also large increases in the proportion of patients diagnosed with early syphilis (from 3.1 to 8.1 %) and in the proportion of newly diagnosed cases that were asymptomatic (from 21 to 85 %). Finally, opt-in, opt-out, and risk-based clinic policies on syphilis screening among HIV-positive MSM were compared in an Australian observational cohort study at sexual health and hospital outpatient clinics. From 2006 to 2010, the proportion of men with at least three syphilis tests per year was highest in clinics with an opt-out policy (48 %), intermediate for clinics with an opt-in policy (39 %), and was considerably lower among men who attended the clinics with risk-based syphilis test policies (8 %) [27].

In Ontario, the majority (86 %) of syphilis tests among people with HIV are ordered by HIV care providers [14], suggesting that HIV clinics are the strategic target to maximize improvements in screening. Using a Markov model, we explored the potential cost-effectiveness of frequent screening of HIV-positive MSM using data from the Ontario HIV Treatment Network (OHTN) Cohort Study. Costs included tests, treatments, and care for neurosyphilis and tertiary syphilis [19]. The model predicted that more frequent screening may not only be cost-effective but cost saving and was the preferred strategy compared to usual care (57 % of the population screening annually) when syphilis incidence was above 0.5 per 100 PY.

Based on promising intervention findings elsewhere [24–27], local syphilis epidemiology in Ontario [14, 22, 30, 31], and current patterns of syphilis testing and diagnosis among HIV-positive MSM in our setting [14, 22], we believe that a clinic-based intervention to routinize syphilis testing with HIV viral loads could be beneficial but that sufficient equipoise exists to warrant a trial with a concurrent control group. The full benefit of such interventions remains uncertain because previous studies were non-randomized trials with historical controls and with short pre- and post-intervention periods. None measured cost-effectiveness, which is essential as health systems must decide on the best use of limited resources. The Enhanced Syphilis Screening among HIV-positive Men (ESSAHM) trial will be the first evaluation of a clinic-based intervention of opt-out syphilis screening among persons with HIV using a randomized, controlled design in multiple clinics.

### Trial aim and objectives

The aim of the ESSAHM trial is to enhance syphilis screening among HIV-positive MSM. We are conducting a clinic-based intervention to incorporate syphilis testing with routine HIV viral load monitoring among HIV-positive men attending four hospital-based HIV outpatient clinics in Toronto and Ottawa, the two largest cities in the province of Ontario. The control condition is the maintenance of current care practices. Specific objectives are to determine to what degree the intervention:

1. Increases the detection rate of untreated syphilis.
2. Increases the proportion of men who undergo syphilis testing at least annually (increased screening coverage)
3. Reduces the interval between syphilis tests (increased screening frequency)
4. Reaches men at highest risk according to sexual behaviors

We hypothesize that the intervention will increase the case detection rate by 75 % or more via an increase in screening coverage to at least 85 % and screening frequency to a median of three tests per person per year. We further hypothesize that increases in screening coverage and frequency will be equivalent among men regardless of sexual behavior characteristics, so that improvements reach men with the highest risk for syphilis infection.

## Methods/design

### Design

**Fig. 1:**
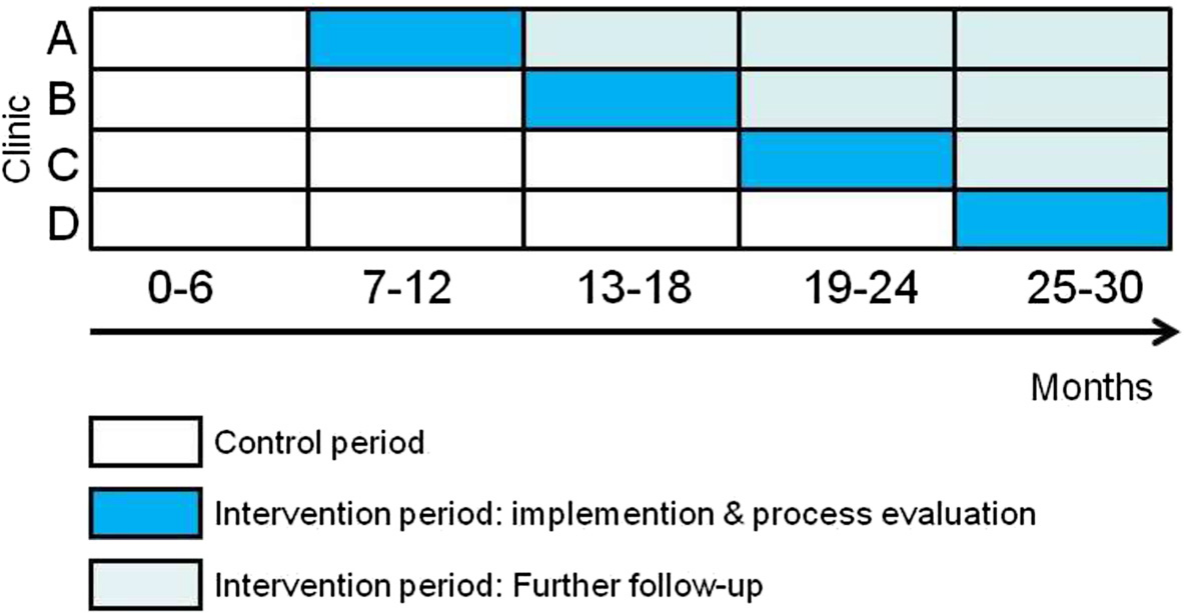
Stepped wedge design for the Enhanced Syphilis Screening among HIV-positive Men (ESSAHM) Trial

We are using a pragmatic, cluster-randomized controlled superiority trial using a stepped wedge design that introduces the intervention stepwise across clinics (Fig. 1). Clinics are randomized to one of four roll-out schedules, each contributing at least one 6-month control period and one 6-month intervention period to the analysis. We selected cluster randomization over individual randomization because the intervention occurs at the clinic level (the “cluster”), even though outcome data is collected at the level of the individual. Unlike parallel trials, the stepped wedge design randomly allocates clusters to the order of implementation, such that all clusters eventually implement the intervention [32–34]. This design is recommended for pragmatic trials when there is a strong belief that the intervention will do more good than harm [34]. The stepped wedge maximizes power compared to parallel cluster randomized trials, requiring fewer clusters [32]. Our approach incorporates a concurrent comparison group, allows for assessment of time trends, and will generate more generalizable results due to its inclusion of multiple clinics.

### Study setting

We targeted settings in Ontario, Canada, where the potential for benefit would be the greatest and the study would be the most feasible. The four participating HIV outpatient clinics are the Ottawa Hospital Immunodeficiency Clinic, St. Michael’s Hospital Positive Care Clinic, Sunnybrook Health Sciences Centre Medical Outpatient Clinic, and Toronto General Hospital Immunodeficiency Clinic. Toronto and Ottawa were selected because these cities have been the epicenters of the Ontario syphilis outbreaks over the past decade [35, 36]. The selected clinics comprise all hospital-based HIV outpatient clinics in these cities and 37 % of all persons receiving HIV care in Ontario who had viral load tests submitted to the centralized provincial laboratory in 2011 (Dr. Robert S. Remis, personal communication, 2013-02-04). Because interpretation of syphilis serology can be challenging, we reasoned that initial assessment of the intervention would be best conducted at clinics with physicians experienced in this area. Based on data from an HIV clinical cohort at these sites, the OHTN Cohort Study [14, 22, 37], an estimated 78 % of persons receiving HIV care at the participating clinics are male, of whom 73 % are MSM. During 2014, only 43 % of MSM at these clinics had at least one syphilis test and the median number of tests was no higher than one, suggesting ample opportunity to increase testing coverage and frequency. Conversely, 99 % of MSM received ≥1 routine HIV viral load test a median of three times per year, providing an estimate of the increase in syphilis testing expected from the intervention (Dr. Sandra Gardner, personal communication, 2015-12-15, Ontario HIV Treatment Network).

### Study population and eligibility criteria

Rather than restricting study activities to MSM, all men receiving HIV care at participating clinics are considered eligible and under observation for the ESSAHM trial, both to simplify study logistics and to minimize the need to disclose sexual status at the time of venipuncture. This obviates the need for repeated detailed assessments of sexual histories [26] and is in keeping with our pragmatic trial design. It may also maximize coverage of men who may not have identified as MSM yet continue to be at risk. Although the rate of syphilis among non-MSM HIV-positive males is reported to be lower than among those identifying as MSM, it is higher than among women, suggesting misclassification of some males as non-MSM [22]. Women are excluded from the study because we have observed no new cases of syphilis among women with HIV [22] nor are such cases being reported in provincial surveillance; from 2002–2014, 99.9 % of all HIV and syphilis co-infected cases in Ontario were male [38].

We will operationalize the study population as all men in HIV care whose viral load monitoring is ordered by physicians at the participating HIV clinics and submitted to the centralized provincial laboratory in Ontario for testing. Men whose viral load monitoring is exclusively done at other sites (e.g., with a family doctor) or via participation in industry-sponsored trials will be considered to be outside of the study population.

### Intervention and control periods

The intervention condition is opt-out standing orders for syphilis serology whenever men undergo routine HIV viral load tests. It is a standard practice for persons in HIV care in these centers to undergo such tests every 3–6 months [28]. The logistics of implementing this change in practice will be tailored to the clinic. Options include pre-printing a checkmark for “syphilis serology” onto existing pre-printed requisitions for routine blood work, addition of the serology request form to the routine blood work package, or programming “syphilis serology” into existing computerized routine order sets. Men can decline syphilis testing or request not to have their data used for the trial at any point throughout the study period. However, we anticipate that compliance will be high.

The control condition is the usual syphilis testing practices carried out by the physicians: testing may be prompted by signs or symptoms, exposure to active cases, men’s disclosure of sexual risk behavior, and physicians’ knowledge and experience diagnosing syphilis.

### Outcomes

The primary outcome is the detection rate of new, previously untreated syphilis cases, specifically acute infectious syphilis. The centralized provincial laboratory in Ontario is the repository of all syphilis tests in Ontario, and these will be accessed to help with syphilis diagnoses. This laboratory uses the following “reverse algorithm” for the serologic diagnosis of syphilis: (1) screen with chemoluminescent (CLIA) test; (2) if CLIA is reactive or indeterminate, additional testing is performed with the rapid plasma reagin (RPR) test as well as the *T. pallidum* particle agglutination (TPPA) test for confirmation of CLIA screening results; (3) if RPR and TPPA are non-reactive or indeterminate, may also include the fluorescent treponemal antibody absorbance (FTA-Abs) test [39]. Based on this approach, a new diagnosis is defined as:

1. Seroconversion with prior negative syphilis serology within the previous 12 months (requires both a reactive syphilis serological screen and a minimum of a second reactive syphilis test using a different serological method, e.g., rapid plasma reagin (RPR)/*T. pallidum* particle agglutination (TPPA); or
2. In men previously diagnosed with syphilis, a fourfold or greater rise in RPR titre from the last titre within the prior 12 months, indicative of re-infection; or
3. Clinical evidence of primary or secondary syphilis together with laboratory confirmation (i.e., direct fluorescent antibody or polymerase chain reaction (PCR) from primary lesions or a positive syphilis screening test together with a second reactive syphilis test using a different serological method).

Diagnoses by clinical (physician ascribed) or laboratory (at least two reactive serological results using different methodologies) criteria which do not meet the above definitions will be considered to be late syphilis (>1 year since infection, non-infectious) or syphilis of unknown duration (if date of infection not known).

Secondary outcomes include screening coverage (proportion of men tested at least once per year) and screening frequency (number of times per year).

### Timeline

**Fig. 2:**
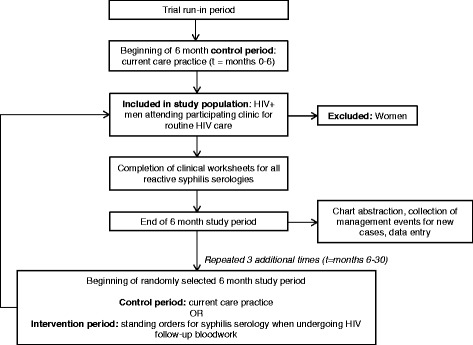
Participant data collection timeline and procedures in the ESSAHM Trial

The data collection period will last a total of 30 months. All clinics began with a trial run-in period in November 2014 during which there was no change in current syphilis test ordering practices. The run-in allowed clinics the opportunity to implement and pilot data collection procedures. Feedback during the run-in was used to modify data collection protocols as needed. After the run-in, clinics transitioned into the first official 6-month control period, starting in February 2015, with no change to syphilis testing practices from the status quo. At the end of this period (August 2015), the intervention was implemented at the first clinic as randomly assigned within the stepped wedge (Fig. 1). At the end of each 6-month period, a new clinic will implement the intervention according to their placement on the wedge. By July 2017, all clinics will have implemented the intervention for a minimum of 6 months. Figure 2 outlines the timeline and procedures for data collection.

### Sample size and power

We used two approaches to anticipate power to detect a ≥75 % increase in our primary outcome, the case detection rate based on the effect size reported in pre-/post-intervention studies [24, 26]. First, using a published method for stepped wedge designs [32], we calculated that we could detect a ≥60 % increase in case detection, assuming an overall baseline case detection rate of 2.5 % [14], a sample size of 2278 men from four sites, the stepped wedge design (Fig. 2), 80 % power, a significance level of 0.05, and a within-clinic intraclass correlation coefficient of 0.004 (OHTN Cohort Study, unpublished data). However, that approach assumes independence of observations across time periods [32]. Following a cohort population such as in the ESSAHM trial violates that assumption because the same subject may appear in more than one time period, introducing within-subject correlation [33, 40, 41]. Therefore, we ran extensive simulations to account for within-clinic and within-subject correlation. Under varying assumptions, the simulations demonstrated that a sample size of 2278 men will provide at least 80 % power to detect a ≥75 % increase in the case detection rate (from 2.5 to 4.4 % or higher). Anticipated power is 90 % or greater to detect changes in both secondary outcomes (screening coverage from 55 to 85 %; screening frequency from one test/year to three tests/year).

### Assignment of intervention

At month 5, we used blinded, random allocation to assign each clinic to the order of implementation on the wedge (Fig. 1). Randomization was performed by a biostatistician who was not a member of the project team and who was blinded to clinic names. Once complete, clinic allocation was unblinded to the project team.

### Data collection and management

Baseline data to characterize the study population include age, number of viral loads ordered in the preceding 12 months, and ever had a requisition for HIV resistance testing/genotyping post initiation of antiretroviral therapy (proxy measure of history of significant virologic failure) and syphilis test history (any past linked syphilis test since 2010 ordered by (i) the clinic and (ii) any Ontario care provider; any past reactive syphilis test since 2010 ordered by (i) the clinic and (ii) any Ontario care provider). Follow-up data will then be appended for each 6-month period: all HIV viral loads and syphilis serology tests ordered by the participating clinics (test dates and results); any viral load ordered by another health care provider in Ontario (month and year of test); and any syphilis serology test ordered by another health care provider in Ontario (month and year of test).

Positive syphilis serology results at the centralized provincial laboratory in Ontario will be supplemented with clinical diagnostic data recorded in patient charts. We developed a standardized clinical worksheet as a tool to aid nurses and physicians in determining which reactive syphilis tests require further follow-up, including a brief, standardized set of questions to facilitate syphilis staging (Online Supplemental Content). The worksheet was pre-tested in all clinics and revised to adequately capture most anticipated situations. In our experience, 40–80 % of syphilis cases can be assigned a stage based on retrospective chart review [8, 22]; the clinical worksheets will further improve this figure. At periodic intervals, an analyst at the centralized provincial laboratory will send to each clinic a batched list of all men receiving HIV care with reactive syphilis results. This will prompt medical chart reviewers at the clinics to enter data from the clinical worksheets and patient charts for all screen test positives into a Microsoft Access database with standardized electronic case report forms. Data integrity mechanisms and reviewer training promote data quality.

Additional clinical, psychosocial, and behavioral data will be available for a subset of men receiving HIV care who are also participants in an ongoing clinical cohort, the OHTN Cohort Study. Its design is fully described elsewhere [14, 22, 37]. Briefly, volunteer participants are attending specialty HIV clinics across Ontario (including all four study sites), are aged 16 years and older, and provide written informed consent. Clinical and sociobehavioral data is collected through clinical chart review, annual interviews, and via record linkage with test data at the centralized provincial laboratory in Ontario. Approximately 40 % of persons receiving HIV care at the four ESSAHM trial clinics are active cohort participants [14, 22, 37]. Participation rates are 20 % higher among MSM [42]. The cohort collects data using medical chart abstractions and an annual interview with a self-administered sexual behavior questionnaire. Measures of sexual behavior in the preceding 3 months include biological sex and number of sexual partners, nature of relationship (regular versus casual), HIV status of partners, and whether the participant engaged in anal intercourse with or without a condom.

### Statistical methods

The analysis will consist of three stages. First, we will assess characteristics of the clinics, men receiving HIV care, and the subgroup of OHTN Cohort Study participants at baseline. Second, we will conduct interim preliminary analysis for assessment of the intervention effect and temporal trends at month 18. Finally, we will conduct comparisons between control and intervention periods using intention-to-treat principles at the end of the study period (month 30), once all clinics have had at least one 6-month intervention period.

Outcomes will be summarized across sites for each time period and between control and intervention time periods within sites. We will use tabular and graphical summaries to describe demographic, behavioral, and clinical characteristics of the participants within and across sites and over time. We will check for delayed treatment effects to evaluate the assumption that the 6-month time periods are long enough to see the full intervention effect.

We will estimate the intervention effect using general linear mixed models (Table 1). Models will take into account within and between site variance components, within-subject correlation, and will have random site effects, a fixed time effect and a fixed effect due to the intervention. With only four clinics in the trial, there may be differences in clinic populations that may confound the intervention effect. Therefore, patient-level covariates will be added to the model (i.e., baseline age, viral load, and syphilis test histories) if necessary to adjust for measurable differences in patient populations across clinics and time periods. In the sub-analysis among men receiving HIV care who are participants of the OHTN Cohort Study, we will additionally include covariates such as sociodemographic characteristics and sexual behaviors if these are empirical confounders of the intervention effect. To determine whether the intervention effects on screening coverage and frequency are equivalent among men across sexual behavior characteristics, we will test for statistical interactions between the fixed effect due to the intervention and sexual behavior variables.

**Table 1:** Variables, measures, and methods of analysis for ESSAHM trial

Variable/outcome	Hypothesis	Outcome measure	Methods of analysis
1) Primary: syphilis case detection	75 % increase over baseline rate	New, previously untreated syphilis case as a binary measure	GLMM models using SAS PROC GLIMMIX with logit function
2) Secondary: a. screening coverage	Increase to 85 % of men testing annually	Tested one or more times in 6-month study period as a binary measure	GLMM models using SAS PROC GLIMMIX with logit function Compare proportion tested and modify to annualized rates
b. screening frequency	Increase to median of 3 tests per person/year	Number of tests per year as Poisson count outcome data	GLMM models using SAS PROC GLIMMIX with log link function
c. reached men at highest risk for syphilis by examining OHTN Cohort Study^a^ subgroup	Increases in screening coverage and frequency (2a&b) occur in the among men at highest risk for syphilis according to sexual behaviors	Outcome measures for screening coverage and screening frequency	GLMM models using SAS PROC GLIMMIX with logit and log link function

*GLMM* general linear mixed models

^a^Ontario HIV Treatment Network Cohort Study, an ongoing clinical HIV cohort taking place at the four participating clinics of the trial [37]. An estimated 40 % of men in HIV care at these clinics are active participants of that cohort

### Additional planned analysis

Using exploratory analyses, we will characterize trends in syphilis testing outside of the confines of the study population and the ESSAHM trial intervention. First, among men in the study population, we will document trends in syphilis testing ordered by providers external to participating clinics according to records at the centralized provincial laboratory in Ontario. Second, we will track rates of syphilis test ordering by participating clinics for clinic patients who are not part of the defined study population. We will also follow public health surveillance case reports to note whether there is ecologic evidence of changes in syphilis diagnosis rates over the study period.

### Process evaluation

We will calculate the proportion of submitted viral loads that have a corresponding requisition for syphilis serology. Qualitatively, debriefing discussions with site clinicians will help identify reasons for any absence of syphilis serology with HIV blood work. Discussion points that arise during debriefing sessions (particularly any of concern to men receiving HIV care) will be brought forth and addressed by the Trial Steering Committee, which includes community representatives. In addition, at the end of the 6-month implementation period at each clinic, we will conduct open-ended qualitative interviews with health care providers about their experiences with the intervention, including perceptions of its utility, convenience, barriers and facilitators to implementation, and opinions on long-term sustainability.

### Economic evaluation

The economic evaluation will be conducted in two steps. Direct and indirect costing of each additional syphilis diagnosis will be estimated using Ontario’s public-payer fee schedules for outpatient services, the Canadian Institute for Health Information cost database by case-mix group for inpatient services, the Ontario Drug Benefit wholesale prices for drug costs, and the wholesale purchase of test kits and technician time at the centralized provincial laboratory in Ontario. Additional costs will be drawn from the published literature [43, 44]. We will calculate costs for the intervention as a whole and individually for each syphilis diagnosis in the intervention and control periods.

In step 1, we will use a net-benefit regression framework to produce the cost-effectiveness acceptability curves, where the *y*-axis represents the probability of the intervention being cost-effective and the *x*-axis represents the willingness to pay [45]. This will be a simple calculation using linear regression with bootstrapping for sensitivity analyses.

In step 2, we will use a dynamic mathematical model of syphilis and HIV transmission among Ontario MSM using the trial data to estimate the projected incremental health benefits (quality of life years gained) per additional cost of identifying new syphilis cases, when examined from a health-provider perspective.

## Ethics

The ESSAHM trial protocol has been approved by the Research Ethics Boards at Ottawa Health Science Network, St. Michael’s Hospital, Sunnybrook Health Sciences Centre, University Health Network, University of Toronto, and Public Health Ontario. The trial is registered at ClinicalTrials.gov (NCT02019043).

As each clinic implements the intervention, multiple strategies will be used to inform males receiving HIV care that the clinic is participating in the ESSAHM trial using an “opt-out” model. All participants will receive information about the study and provide verbal consent. However, written consent is not required, in keeping with the Canadian Tri-Council Policy Statement (TCPS2) Articles 3.7 to 3.11 and the Ontario Personal Health Information Protection Act 44, 3c and d [46]. Health care providers will discuss the trial and circulate posters and brochures informing men about the study, the right to opt-out and how to opt-out. At any time, men receiving HIV care can opt-out of syphilis testing and/or refuse to have their clinical data used for the trial by simply telling their health care provider that they wish not to be included.

The risks associated with the ESSAHM trial are modest. First, although syphilis testing requires the additional collection of 7 mL of blood, there will be negligible additional health risk to men receiving HIV care because separate venipuncture is not required. Second, there is a theoretical risk of over-treatment due to false positive syphilis serology results, particularly for low-risk men. However, the risk of over-treatment will be mitigated by clinical judgment that takes into account self-reported sexual behavior and past history of syphilis test results in making the final determination whether to treat, and more frequent syphilis testing will help clarify syphilis history. Further, a syphilis diagnosis may cause men stress, anxiety, and fear of stigmatization; such risks will be mitigated via appropriate counseling by physicians and the high success rate of syphilis treatments.

### Data confidentiality and access

At the clinics, participant information will either be securely stored in patient charts or locked file cabinets in areas with limited access. To maintain confidentiality of men in the study, all will be given a “Case ID” and no personal identifying information will be entered into the study database. Extractions at the centralized provincial laboratory in Ontario will be performed by authorized personnel and stored in a secure network environment with limited access. Information sent to the clinics will be faxed using a secure fax system. When data are transferred from the laboratory and clinics to the investigating team for analysis, they will be de-identified, encrypted, and sent through a secure server. Data will be stored on password-protected computers located in a secure server environment. They will remain encrypted when not in use. Access will be restricted to members of the study team who will sign confidentiality agreements. Data will never be reported in groups of less than five individuals in any cell or data block to further guard against the inadvertent disclosure of identities.

### Dissemination

Results of the ESSAHM trial will be reported according to a pre-determined publication policy approved by all members of the research team. Study results will be disseminated via scientific conferences and publications; presentations to public health working groups, health care providers, and community symposia for men living with HIV; and meetings with decision-makers.

## Trial status

The ESSAHM trial run-in period began in November 2014, and the official start date was February 2015. Data collection will continue until July 2017.

## Discussion

Syphilis diagnoses occur at a rate of 3.3 per 100 PY among HIV-positive men in Ontario [14], yet testing in this population remains below recommended guidelines [6, 17, 18]. Routinizing syphilis testing with regular HIV blood work has shown potential to improve case detection in uncontrolled studies. However, a randomized, controlled, pragmatic, multi-center trial is needed to firmly establish the public health impact and cost-effectiveness of this approach. The ESSAHM trial design maximizes rigor within the practical constraints of our setting. We opted for a pragmatic approach consistent with “real life” conditions if the centralized provincial laboratory in Ontario were to routinize syphilis testing with viral loads at regional or provincial levels.

There are potential threats to validity for which we will remain vigilant. As of June 2015, syphilis case reporting remained high in Ontario [38]. Nevertheless, secular trends in syphilis testing and/or incidence may result in the intervention being more or less effective than anticipated. In an era where viral load monitoring may become less frequent for persons in HIV care who are on a stable, fully suppressive antiretroviral treatment regimen [28], the realized benefits of an opt-out screening program attached to HIV viral load testing may lessen over time. The stepped wedge design will allow for assessment of underlying time trends [34] which should mitigate bias due to secular changes. As for other screening interventions, case detection may be initially high due to diagnosis of prevalent cases but may then decrease as only incident cases remain; we will be able to quantify such trends for those clinics randomized to earlier implementation of the intervention. The trial involves investigators who are practicing clinicians at the participating sites, which could introduce a Hawthorne effect; nevertheless, each clinic also includes several non-study physicians. Although it is a multi-site trial, it involves only four clinics and excludes persons receiving HIV care whose viral loads are monitored elsewhere. Detailed description of the trial population, setting, and local contexts will allow for future assessments regarding generalizability to other populations.

Lessons learned from the ESSAHM trial will guide policy and practice decisions regarding scale-up in these and other HIV clinics in Ontario and at the centralized provincial laboratory. Members of the team (DHST, PM, SM) are also conducting studies to determine the most appropriate management strategies for syphilis/HIV co-infected persons in HIV care. Results will contribute to comprehensive, evidence-based recommendations on policies, practices, and strategies to reduce syphilis burden among MSM. Moreover, knowledge gained on the potential benefit of syphilis screening interventions from the ESSAHM trial may be applicable to other sexually transmitted and blood-borne infections that pose risk for men receiving HIV care, including chlamydia, gonorrhea, and hepatitis viruses.

## Abbreviations

CLIA: chemoluminescent
ESSAHM: Enhanced Syphilis Screening among HIV-positive Men
FTA-Abs: fluorescent treponemal antibody absorbance
HIV: human immunodeficiency virus
MSM: men who have sex with men
OHTN: Ontario HIV Treatment Network
PCR: polymerase chain reaction
RPR: rapid plasma reagin
STI: sexually transmitted infection
TPPA: *Treponema pallidum* particle agglutination
